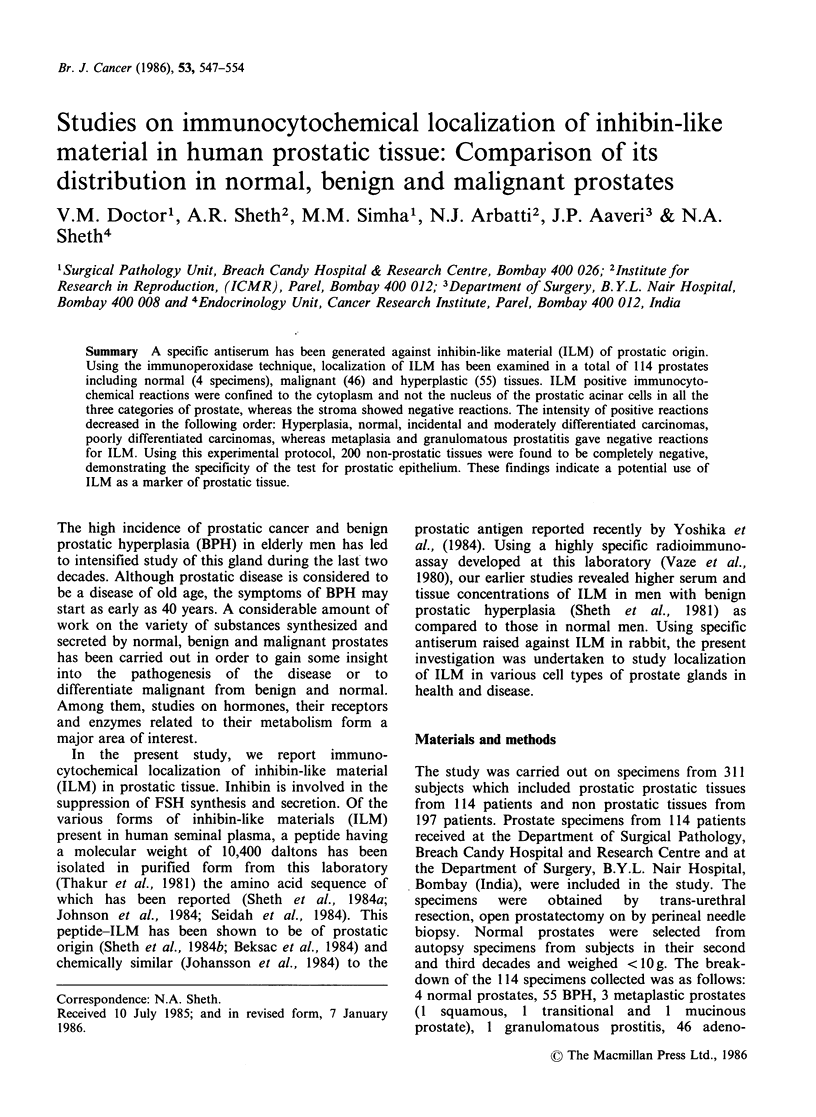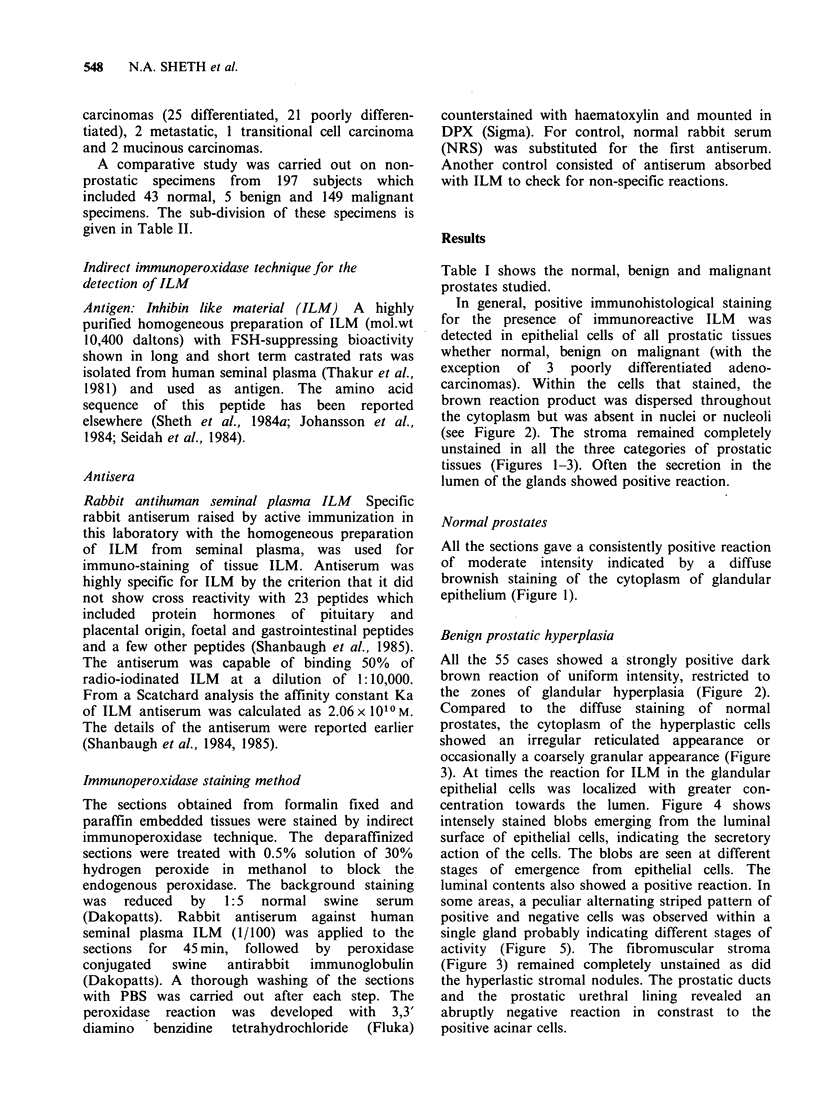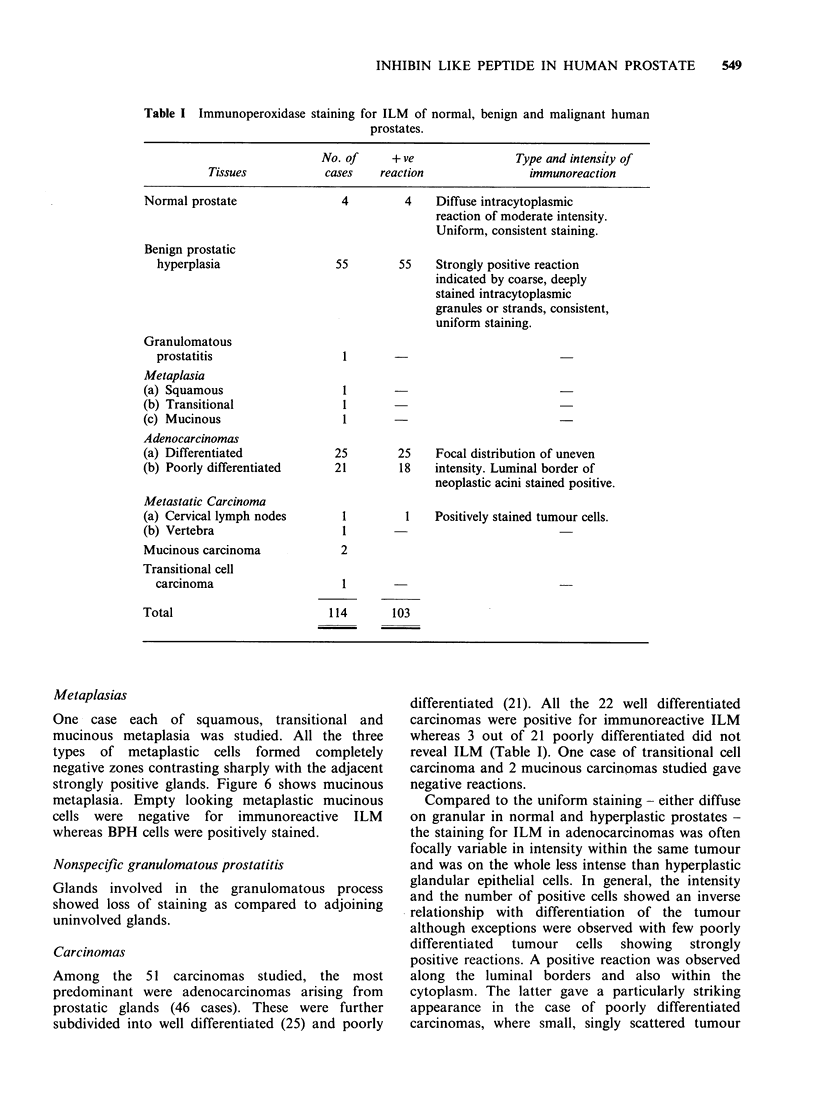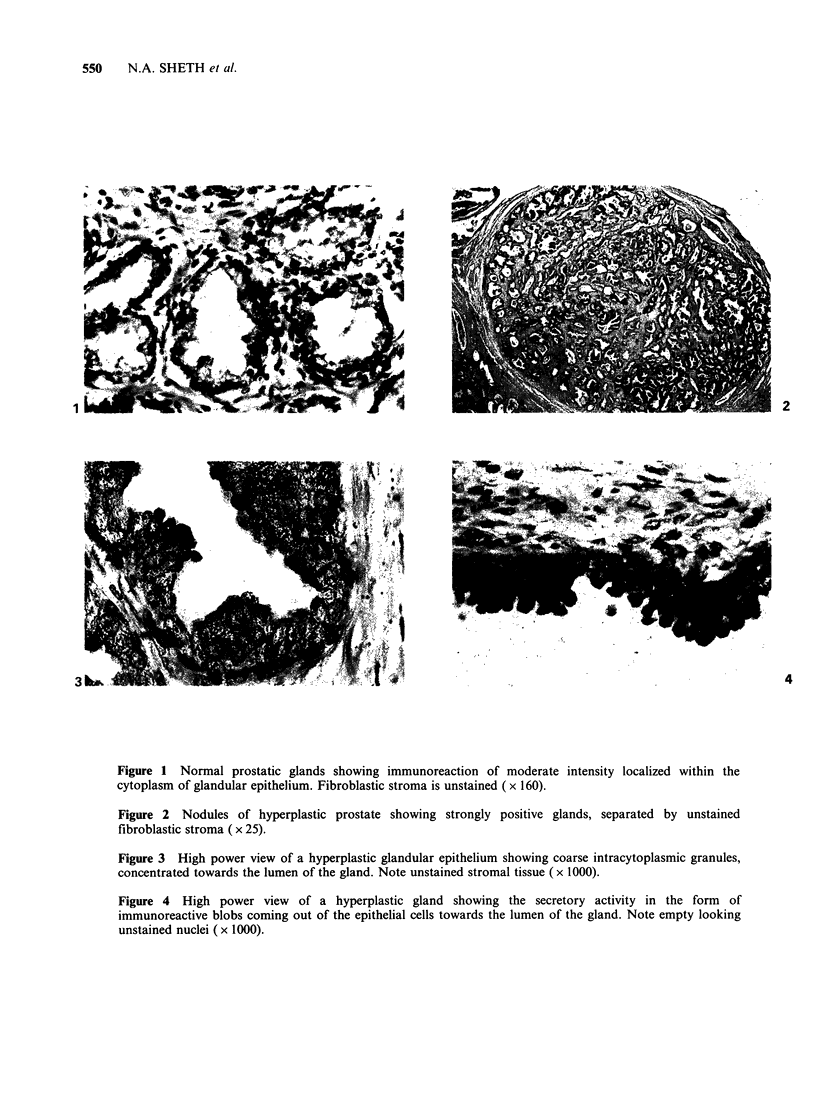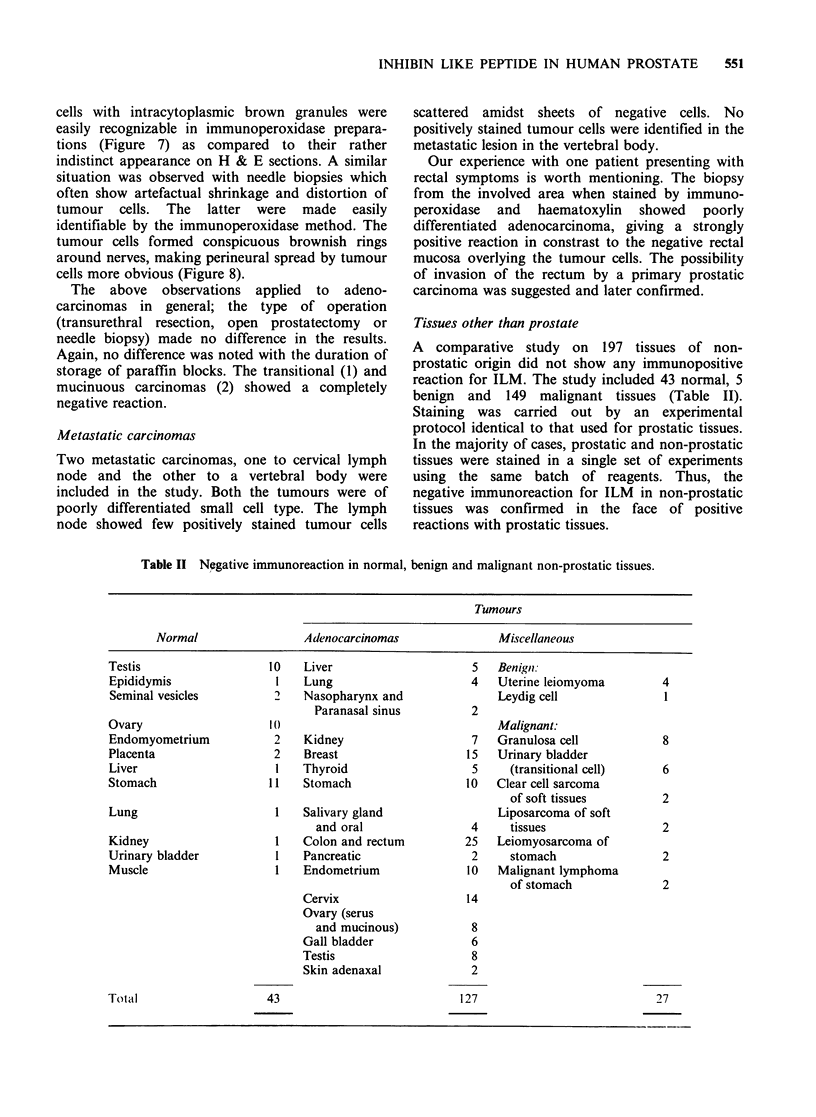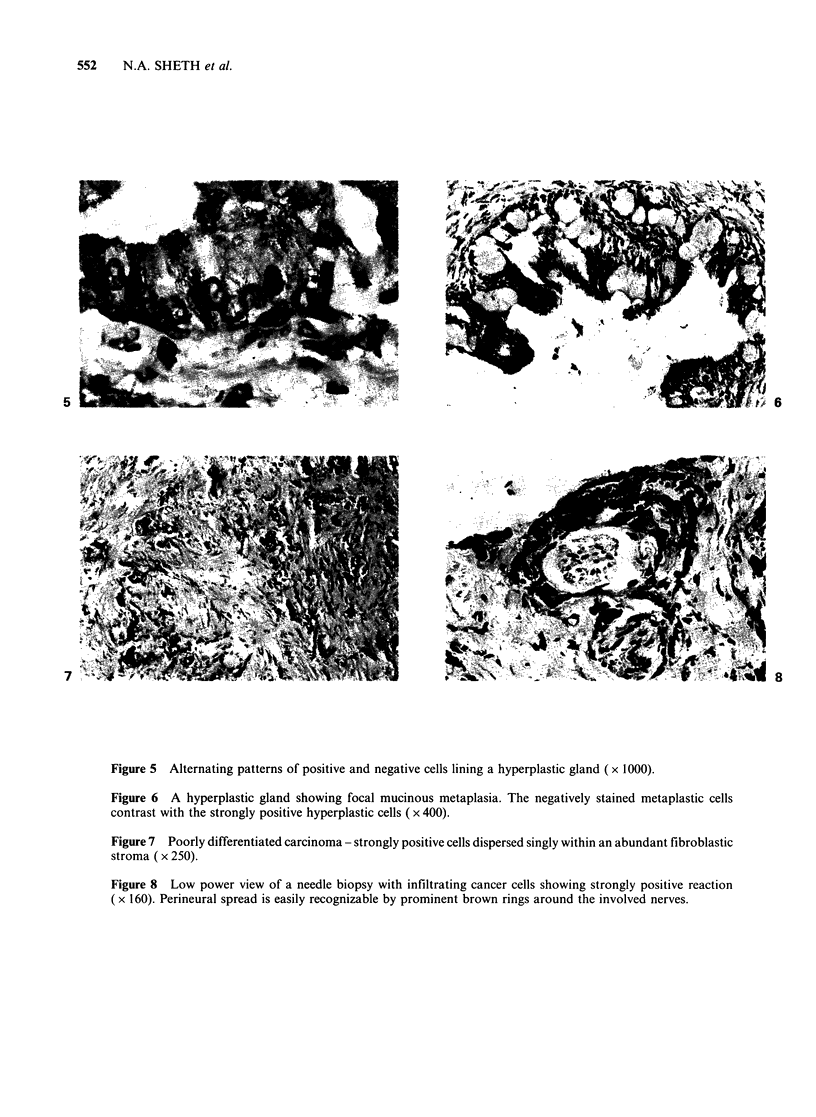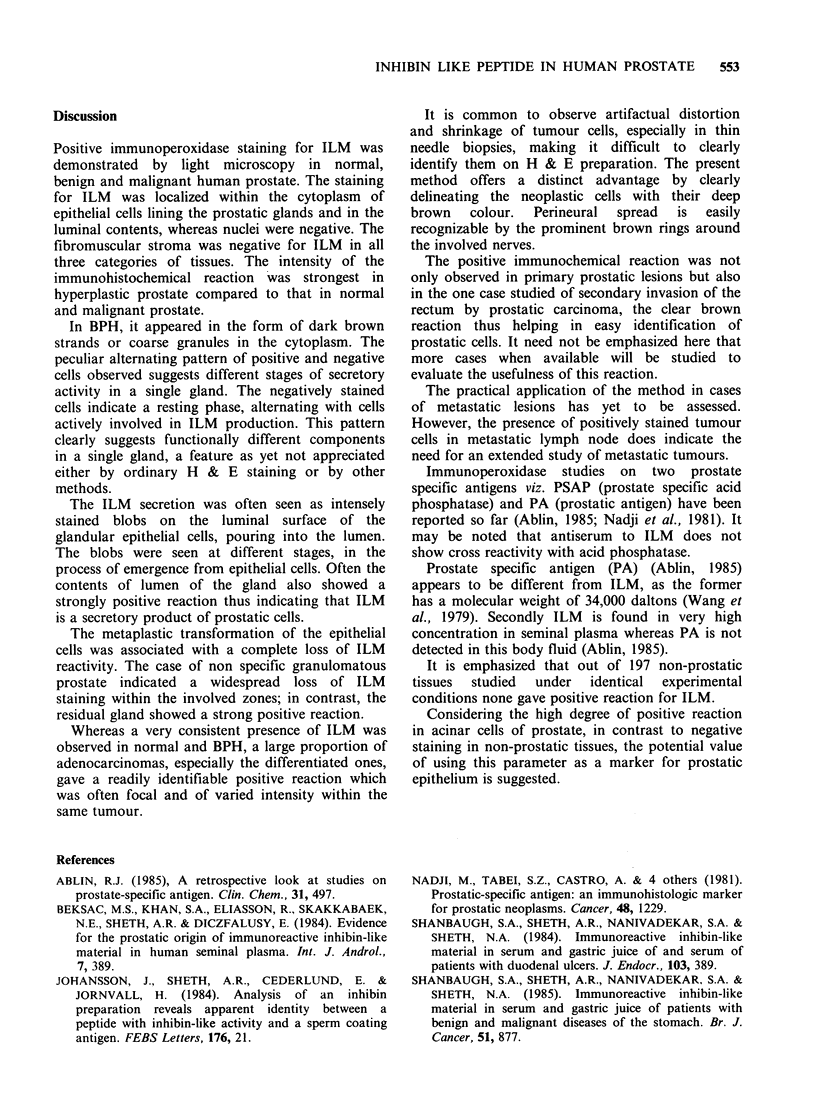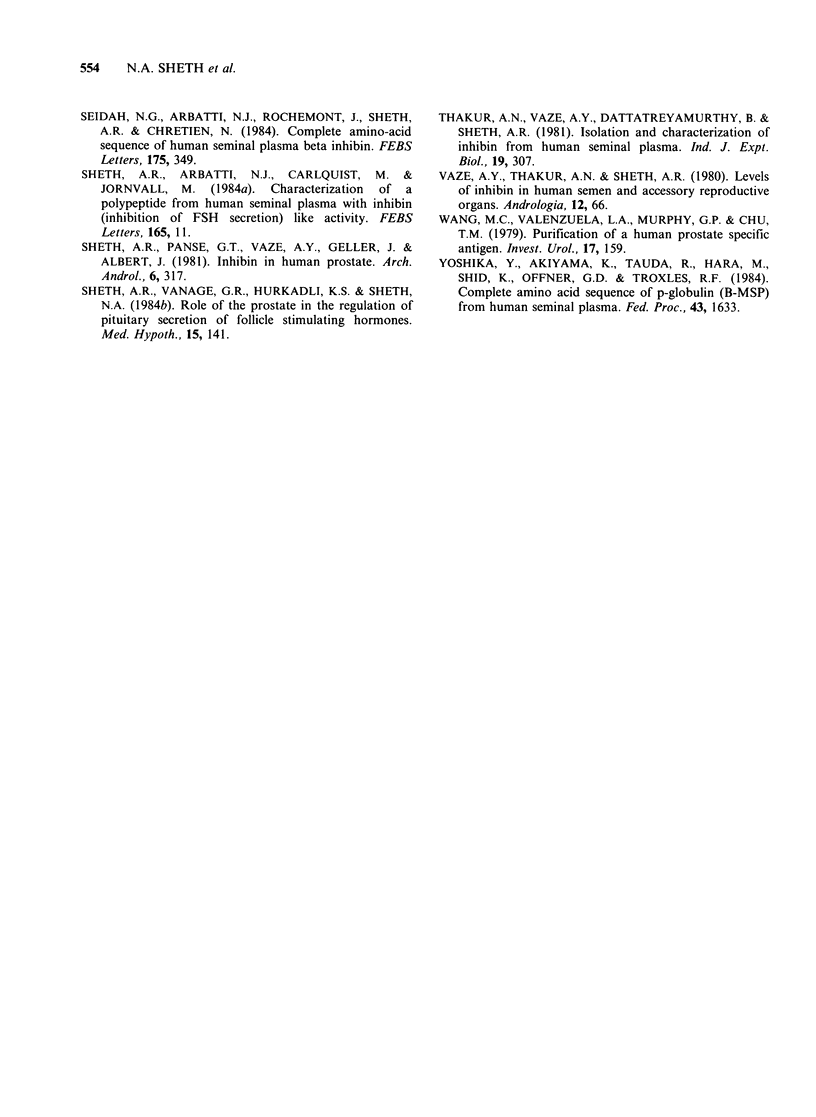# Studies on immunocytochemical localization of inhibin-like material in human prostatic tissue: comparison of its distribution in normal, benign and malignant prostates.

**DOI:** 10.1038/bjc.1986.85

**Published:** 1986-04

**Authors:** V. M. Doctor, A. R. Sheth, M. M. Simha, N. J. Arbatti, J. P. Aaveri, N. A. Sheth

## Abstract

**Images:**


*Br. J. Cancer* (1986), **53**, 547–554

### Studies on immunocytochemical localization of inhibin-like material in human prostatic tissue: Comparison of its distribution in normal, benign and malignant prostates

V.M. Doctor[1], A.R. Sheth[2], M.M. Simha[1], N.J. Arbatti[2], J.P. Aaveri[3] & N.A. Sheth[4]

[1]*Surgical Pathology Unit, Breach Candy Hospital & Research Centre, Bombay 400 026;* [2]*Institute for Research in Reproduction, (ICMR), Parel, Bombay 400 012;* [3]*Department of Surgery, B.Y.L. Nair Hospital, Bombay 400 008 and* [4]*Endocrinology Unit, Cancer Research Institute, Parel, Bombay 400 012, India*

**Summary** A specific antiserum has been generated against inhibin-like material (ILM) of prostatic origin. Using the immunoperoxidase technique, localization of ILM has been examined in a total of 114 prostates including normal (4 specimens), malignant (46) and hyperplastic (55) tissues. ILM positive immunocytochemical reactions were confined to the cytoplasm and not the nucleus of the prostatic acinar cells in all the three categories of prostate, whereas the stroma showed negative reactions. The intensity of positive reactions decreased in the following order: Hyperplasia, normal, incidental and moderately differentiated carcinomas, poorly differentiated carcinomas, whereas metaplasia and granulomatous prostatitis gave negative reactions for ILM. Using this experimental protocol, 200 non-prostatic tissues were found to be completely negative, demonstrating the specificity of the test for prostatic epithelium. These findings indicate a potential use of ILM as a marker of prostatic tissue.

The high incidence of prostatic cancer and benign prostatic hyperplasia (BPH) in elderly men has led to intensified study of this gland during the last two decades. Although prostatic disease is considered to be a disease of old age, the symptoms of BPH may start as early as 40 years. A considerable amount of work on the variety of substances synthesized and secreted by normal, benign and malignant prostates has been carried out in order to gain some insight into the pathogenesis of the disease or to differentiate malignant from benign and normal. Among them, studies on hormones, their receptors and enzymes related to their metabolism form a major area of interest.

In the present study, we report immunocytochemical localization of inhibin-like material (ILM) in prostatic tissue. Inhibin is involved in the suppression of FSH synthesis and secretion. Of the various forms of inhibin-like materials (ILM) present in human seminal plasma, a peptide having a molecular weight of 10,400 daltons has been isolated in purified form from this laboratory (Thakur *et al.*, 1981) the amino acid sequence of which has been reported (Sheth *et al.*, 1984*a*; Johnson *et al.*, 1984; Seidah *et al.*, 1984). This peptide–ILM has been shown to be of prostatic origin (Sheth *et al.*, 1984*b*; Beksac *et al.*, 1984) and chemically similar (Johansson *et al.*, 1984) to the prostatic antigen reported recently by Yoshika *et al.*, (1984). Using a highly specific radioimmunoassay developed at this laboratory (Vaze *et al.*, 1980), our earlier studies revealed higher serum and tissue concentrations of ILM in men with benign prostatic hyperplasia (Sheth *et al.*, 1981) as compared to those in normal men. Using specific antiserum raised against ILM in rabbit, the present investigation was undertaken to study localization of ILM in various cell types of prostate glands in health and disease.

### Materials and methods

The study was carried out on specimens from 311 subjects which included prostatic prostatic tissues from 114 patients and non prostatic tissues from 197 patients. Prostate specimens from 114 patients received at the Department of Surgical Pathology, Breach Candy Hospital and Research Centre and at the Department of Surgery, B.Y.L. Nair Hospital, Bombay (India), were included in the study. The specimens were obtained by trans-urethral resection, open prostatectomy on by perineal needle biopsy. Normal prostates were selected from autopsy specimens from subjects in their second and third decades and weighed <10 g. The breakdown of the 114 specimens collected was as follows: 4 normal prostates, 55 BPH, 3 metaplastic prostates (1 squamous, 1 transitional and 1 mucinous prostate), 1 granulomatous prostitis, 46 adeno-

Correspondence: N.A. Sheth.
Received 10 July 1985; and in revised form, 7 January 1986.

© The Macmillan Press Ltd., 1986

carcinomas (25 differentiated, 21 poorly differentiated), 2 metastatic, 1 transitional cell carcinoma and 2 mucinous carcinomas.

A comparative study was carried out on non-prostatic specimens from 197 subjects which included 43 normal, 5 benign and 149 malignant specimens. The sub-division of these specimens is given in Table II.

*Indirect immunoperoxidase technique for the detection of ILM*

*Antigen: Inhibin like material (ILM)* A highly purified homogeneous preparation of ILM (mol.wt 10,400 daltons) with FSH-suppressing bioactivity shown in long and short term castrated rats was isolated from human seminal plasma (Thakur *et al.*, 1981) and used as antigen. The amino acid sequence of this peptide has been reported elsewhere (Sheth *et al.*, 1984*a*; Johansson *et al.*, 1984; Seidah *et al.*, 1984).

*Antisera*

*Rabbit antihuman seminal plasma ILM* Specific rabbit antiserum raised by active immunization in this laboratory with the homogeneous preparation of ILM from seminal plasma, was used for immuno-staining of tissue ILM. Antiserum was highly specific for ILM by the criterion that it did not show cross reactivity with 23 peptides which included protein hormones of pituitary and placental origin, foetal and gastrointestinal peptides and a few other peptides (Shanbaugh *et al.*, 1985). The antiserum was capable of binding 50% of radio-iodinated ILM at a dilution of 1:10,000. From a Scatchard analysis the affinity constant Ka of ILM antiserum was calculated as $2.06 \times 10^{10}$ M. The details of the antiserum were reported earlier (Shanbaugh *et al.*, 1984, 1985).

*Immunoperoxidase staining method*

The sections obtained from formalin fixed and paraffin embedded tissues were stained by indirect immunoperoxidase technique. The deparaffinized sections were treated with 0.5% solution of 30% hydrogen peroxide in methanol to block the endogenous peroxidase. The background staining was reduced by 1:5 normal swine serum (Dakopatts). Rabbit antiserum against human seminal plasma ILM (1/100) was applied to the sections for 45 min, followed by peroxidase conjugated swine antirabbit immunoglobulin (Dakopatts). A thorough washing of the sections with PBS was carried out after each step. The peroxidase reaction was developed with 3,3′ diamino benzidine tetrahydrochloride (Fluka) counterstained with haematoxylin and mounted in DPX (Sigma). For control, normal rabbit serum (NRS) was substituted for the first antiserum. Another control consisted of antiserum absorbed with ILM to check for non-specific reactions.

### Results

Table I shows the normal, benign and malignant prostates studied.

In general, positive immunohistological staining for the presence of immunoreactive ILM was detected in epithelial cells of all prostatic tissues whether normal, benign on malignant (with the exception of 3 poorly differentiated adenocarcinomas). Within the cells that stained, the brown reaction product was dispersed throughout the cytoplasm but was absent in nuclei or nucleoli (see Figure 2). The stroma remained completely unstained in all the three categories of prostatic tissues (Figures 1–3). Often the secretion in the lumen of the glands showed positive reaction.

*Normal prostates*

All the sections gave a consistently positive reaction of moderate intensity indicated by a diffuse brownish staining of the cytoplasm of glandular epithelium (Figure 1).

*Benign prostatic hyperplasia*

All the 55 cases showed a strongly positive dark brown reaction of uniform intensity, restricted to the zones of glandular hyperplasia (Figure 2). Compared to the diffuse staining of normal prostates, the cytoplasm of the hyperplastic cells showed an irregular reticulated appearance or occasionally a coarsely granular appearance (Figure 3). At times the reaction for ILM in the glandular epithelial cells was localized with greater concentration towards the lumen. Figure 4 shows intensely stained blobs emerging from the luminal surface of epithelial cells, indicating the secretory action of the cells. The blobs are seen at different stages of emergence from epithelial cells. The luminal contents also showed a positive reaction. In some areas, a peculiar alternating striped pattern of positive and negative cells was observed within a single gland probably indicating different stages of activity (Figure 5). The fibromuscular stroma (Figure 3) remained completely unstained as did the hyperlastic stromal nodules. The prostatic ducts and the prostatic urethral lining revealed an abruptly negative reaction in constrast to the positive acinar cells.

**Table I** Immunoperoxidase staining for ILM of normal, benign and malignant human prostates.

| *Tissues* | *No. of cases* | *+ve reaction* | *Type and intensity of immunoreaction* |
|---|---|---|---|
| Normal prostate | 4 | 4 | Diffuse intracytoplasmic reaction of moderate intensity. Uniform, consistent staining. |
| Benign prostatic hyperplasia | 55 | 55 | Strongly positive reaction indicated by coarse, deeply stained intracytoplasmic granules or strands, consistent, uniform staining. |
| Granulomatous prostatitis | 1 | — | — |
| *Metaplasia* | | | |
| (a) Squamous | 1 | — | — |
| (b) Transitional | 1 | — | — |
| (c) Mucinous | 1 | — | — |
| *Adenocarcinomas* | | | |
| (a) Differentiated | 25 | 25 | Focal distribution of uneven intensity. Luminal border of neoplastic acini stained positive. |
| (b) Poorly differentiated | 21 | 18 | |
| *Metastatic Carcinoma* | | | |
| (a) Cervical lymph nodes | 1 | 1 | Positively stained tumour cells. |
| (b) Vertebra | 1 | — | — |
| Mucinous carcinoma | 2 | | |
| Transitional cell carcinoma | 1 | — | — |
| Total | 114 | 103 | |

### *Metaplasias*

One case each of squamous, transitional and mucinous metaplasia was studied. All the three types of metaplastic cells formed completely negative zones contrasting sharply with the adjacent strongly positive glands. Figure 6 shows mucinous metaplasia. Empty looking metaplastic mucinous cells were negative for immunoreactive ILM whereas BPH cells were positively stained.

### *Nonspecific granulomatous prostatitis*

Glands involved in the granulomatous process showed loss of staining as compared to adjoining uninvolved glands.

### *Carcinomas*

Among the 51 carcinomas studied, the most predominant were adenocarcinomas arising from prostatic glands (46 cases). These were further subdivided into well differentiated (25) and poorly differentiated (21). All the 22 well differentiated carcinomas were positive for immunoreactive ILM whereas 3 out of 21 poorly differentiated did not reveal ILM (Table I). One case of transitional cell carcinoma and 2 mucinous carcinomas studied gave negative reactions.

Compared to the uniform staining – either diffuse on granular in normal and hyperplastic prostates – the staining for ILM in adenocarcinomas was often focally variable in intensity within the same tumour and was on the whole less intense than hyperplastic glandular epithelial cells. In general, the intensity and the number of positive cells showed an inverse relationship with differentiation of the tumour although exceptions were observed with few poorly differentiated tumour cells showing strongly positive reactions. A positive reaction was observed along the luminal borders and also within the cytoplasm. The latter gave a particularly striking appearance in the case of poorly differentiated carcinomas, where small, singly scattered tumour

1

2

3

4

**Figure 1** Normal prostatic glands showing immunoreaction of moderate intensity localized within the cytoplasm of glandular epithelium. Fibroblastic stroma is unstained ( × 160).

**Figure 2** Nodules of hyperplastic prostate showing strongly positive glands, separated by unstained fibroblastic stroma ( × 25).

**Figure 3** High power view of a hyperplastic glandular epithelium showing coarse intracytoplasmic granules, concentrated towards the lumen of the gland. Note unstained stromal tissue ( × 1000).

**Figure 4** High power view of a hyperplastic gland showing the secretory activity in the form of immunoreactive blobs coming out of the epithelial cells towards the lumen of the gland. Note empty looking unstained nuclei ( × 1000).

cells with intracytoplasmic brown granules were easily recognizable in immunoperoxidase preparations (Figure 7) as compared to their rather indistinct appearance on H & E sections. A similar situation was observed with needle biopsies which often show artefactual shrinkage and distortion of tumour cells. The latter were made easily identifiable by the immunoperoxidase method. The tumour cells formed conspicuous brownish rings around nerves, making perineural spread by tumour cells more obvious (Figure 8).

The above observations applied to adenocarcinomas in general; the type of operation (transurethral resection, open prostatectomy or needle biopsy) made no difference in the results. Again, no difference was noted with the duration of storage of paraffin blocks. The transitional (1) and mucinuous carcinomas (2) showed a completely negative reaction.

*Metastatic carcinomas*

Two metastatic carcinomas, one to cervical lymph node and the other to a vertebral body were included in the study. Both the tumours were of poorly differentiated small cell type. The lymph node showed few positively stained tumour cells scattered amidst sheets of negative cells. No positively stained tumour cells were identified in the metastatic lesion in the vertebral body.

Our experience with one patient presenting with rectal symptoms is worth mentioning. The biopsy from the involved area when stained by immunoperoxidase and haematoxylin showed poorly differentiated adenocarcinoma, giving a strongly positive reaction in constrast to the negative rectal mucosa overlying the tumour cells. The possibility of invasion of the rectum by a primary prostatic carcinoma was suggested and later confirmed.

*Tissues other than prostate*

A comparative study on 197 tissues of non-prostatic origin did not show any immunopositive reaction for ILM. The study included 43 normal, 5 benign and 149 malignant tissues (Table II). Staining was carried out by an experimental protocol identical to that used for prostatic tissues. In the majority of cases, prostatic and non-prostatic tissues were stained in a single set of experiments using the same batch of reagents. Thus, the negative immunoreaction for ILM in non-prostatic tissues was confirmed in the face of positive reactions with prostatic tissues.

**Table II** Negative immunoreaction in normal, benign and malignant non-prostatic tissues.

| | | *Tumours* | | | |
|---|---|---|---|---|---|
| *Normal* | | *Adenocarcinomas* | | *Miscellaneous* | |
| Testis | 10 | Liver | 5 | *Benign:* | |
| Epididymis | 1 | Lung | 4 | Uterine leiomyoma | 4 |
| Seminal vesicles | 2 | Nasopharynx and Paranasal sinus | 2 | Leydig cell | 1 |
| Ovary | 10 | | | *Malignant:* | |
| Endomyometrium | 2 | Kidney | 7 | Granulosa cell | 8 |
| Placenta | 2 | Breast | 15 | Urinary bladder (transitional cell) | 6 |
| Liver | 1 | Thyroid | 5 | | |
| Stomach | 11 | Stomach | 10 | Clear cell sarcoma of soft tissues | 2 |
| Lung | 1 | Salivary gland and oral | 4 | Liposarcoma of soft tissues | 2 |
| Kidney | 1 | Colon and rectum | 25 | Leiomyosarcoma of stomach | 2 |
| Urinary bladder | 1 | Pancreatic | 2 | | |
| Muscle | 1 | Endometrium | 10 | Malignant lymphoma of stomach | 2 |
| | | Cervix | 14 | | |
| | | Ovary (serus and mucinous) | 8 | | |
| | | Gall bladder | 6 | | |
| | | Testis | 8 | | |
| | | Skin adenaxal | 2 | | |
| Total | 43 | | 127 | | 27 |

5

6

7

8

**Figure 5** Alternating patterns of positive and negative cells lining a hyperplastic gland ( × 1000).

**Figure 6** A hyperplastic gland showing focal mucinous metaplasia. The negatively stained metaplastic cells contrast with the strongly positive hyperplastic cells ( × 400).

**Figure 7** Poorly differentiated carcinoma – strongly positive cells dispersed singly within an abundant fibroblastic stroma ( × 250).

**Figure 8** Low power view of a needle biopsy with infiltrating cancer cells showing strongly positive reaction ( × 160). Perineural spread is easily recognizable by prominent brown rings around the involved nerves.

### Discussion

Positive immunoperoxidase staining for ILM was demonstrated by light microscopy in normal, benign and malignant human prostate. The staining for ILM was localized within the cytoplasm of epithelial cells lining the prostatic glands and in the luminal contents, whereas nuclei were negative. The fibromuscular stroma was negative for ILM in all three categories of tissues. The intensity of the immunohistochemical reaction was strongest in hyperplastic prostate compared to that in normal and malignant prostate.

In BPH, it appeared in the form of dark brown strands or coarse granules in the cytoplasm. The peculiar alternating pattern of positive and negative cells observed suggests different stages of secretory activity in a single gland. The negatively stained cells indicate a resting phase, alternating with cells actively involved in ILM production. This pattern clearly suggests functionally different components in a single gland, a feature as yet not appreciated either by ordinary H & E staining or by other methods.

The ILM secretion was often seen as intensely stained blobs on the luminal surface of the glandular epithelial cells, pouring into the lumen. The blobs were seen at different stages, in the process of emergence from epithelial cells. Often the contents of lumen of the gland also showed a strongly positive reaction thus indicating that ILM is a secretory product of prostatic cells.

The metaplastic transformation of the epithelial cells was associated with a complete loss of ILM reactivity. The case of non specific granulomatous prostate indicated a widespread loss of ILM staining within the involved zones; in contrast, the residual gland showed a strong positive reaction.

Whereas a very consistent presence of ILM was observed in normal and BPH, a large proportion of adenocarcinomas, especially the differentiated ones, gave a readily identifiable positive reaction which was often focal and of varied intensity within the same tumour.

It is common to observe artifactual distortion and shrinkage of tumour cells, especially in thin needle biopsies, making it difficult to clearly identify them on H & E preparation. The present method offers a distinct advantage by clearly delineating the neoplastic cells with their deep brown colour. Perineural spread is easily recognizable by the prominent brown rings around the involved nerves.

The positive immunochemical reaction was not only observed in primary prostatic lesions but also in the one case studied of secondary invasion of the rectum by prostatic carcinoma, the clear brown reaction thus helping in easy identification of prostatic cells. It need not be emphasized here that more cases when available will be studied to evaluate the usefulness of this reaction.

The practical application of the method in cases of metastatic lesions has yet to be assessed. However, the presence of positively stained tumour cells in metastatic lymph node does indicate the need for an extended study of metastatic tumours.

Immunoperoxidase studies on two prostate specific antigens *viz.* PSAP (prostate specific acid phosphatase) and PA (prostatic antigen) have been reported so far (Ablin, 1985; Nadji *et al.*, 1981). It may be noted that antiserum to ILM does not show cross reactivity with acid phosphatase.

Prostate specific antigen (PA) (Ablin, 1985) appears to be different from ILM, as the former has a molecular weight of 34,000 daltons (Wang *et al.*, 1979). Secondly ILM is found in very high concentration in seminal plasma whereas PA is not detected in this body fluid (Ablin, 1985).

It is emphasized that out of 197 non-prostatic tissues studied under identical experimental conditions none gave positive reaction for ILM.

Considering the high degree of positive reaction in acinar cells of prostate, in contrast to negative staining in non-prostatic tissues, the potential value of using this parameter as a marker for prostatic epithelium is suggested.

### References

ABLIN, R.J. (1985), A retrospective look at studies on prostate-specific antigen. *Clin. Chem.*, **31**, 497.

BEKSAC, M.S., KHAN, S.A., ELIASSON, R., SKAKKABAEK, N.E., SHETH, A.R. & DICZFALUSY, E. (1984). Evidence for the prostatic origin of immunoreactive inhibin-like material in human seminal plasma. *Int. J. Androl.*, **7**, 389.

JOHANSSON, J., SHETH, A.R., CEDERLUND, E. & JORNVALL, H. (1984). Analysis of an inhibin preparation reveals apparent identity between a peptide with inhibin-like activity and a sperm coating antigen. *FEBS Letters*, **176**, 21.

NADJI, M., TABEI, S.Z., CASTRO, A. & 4 others (1981). Prostatic-specific antigen: an immunohistologic marker for prostatic neoplasms. *Cancer*, **48**, 1229.

SHANBAUGH, S.A., SHETH, A.R., NANIVADEKAR, S.A. & SHETH, N.A. (1984). Immunoreactive inhibin-like material in serum and gastric juice of and serum of patients with duodenal ulcers. *J. Endocr.*, **103**, 389.

SHANBAUGH, S.A., SHETH, A.R., NANIVADEKAR, S.A. & SHETH, N.A. (1985). Immunoreactive inhibin-like material in serum and gastric juice of patients with benign and malignant diseases of the stomach. *Br. J. Cancer*, **51**, 877.

SEIDAH, N.G., ARBATTI, N.J., ROCHEMONT, J., SHETH, A.R. & CHRETIEN, N. (1984). Complete amino-acid sequence of human seminal plasma beta inhibin. *FEBS Letters*, **175**, 349.

SHETH, A.R., ARBATTI, N.J., CARLQUIST, M. & JORNVALL, M. (1984*a*). Characterization of a polypeptide from human seminal plasma with inhibin (inhibition of FSH secretion) like activity. *FEBS Letters*, **165**, 11.

SHETH, A.R., PANSE, G.T., VAZE, A.Y., GELLER, J. & ALBERT, J. (1981). Inhibin in human prostate. *Arch. Androl.*, **6**, 317.

SHETH, A.R., VANAGE, G.R., HURKADLI, K.S. & SHETH, N.A. (1984*b*). Role of the prostate in the regulation of pituitary secretion of follicle stimulating hormones. *Med. Hypoth.*, **15**, 141.

THAKUR, A.N., VAZE, A.Y., DATTATREYAMURTHY, B. & SHETH, A.R. (1981). Isolation and characterization of inhibin from human seminal plasma. *Ind. J. Expt. Biol.*, **19**, 307.

VAZE, A.Y., THAKUR, A.N. & SHETH, A.R. (1980). Levels of inhibin in human semen and accessory reproductive organs. *Andrologia*, **12**, 66.

WANG, M.C., VALENZUELA, L.A., MURPHY, G.P. & CHU, T.M. (1979). Purification of a human prostate specific antigen. *Invest. Urol.*, **17**, 159.

YOSHIKA, Y., AKIYAMA, K., TAUDA, R., HARA, M., SHID, K., OFFNER, G.D. & TROXLES, R.F. (1984). Complete amino acid sequence of p-globulin (B-MSP) from human seminal plasma. *Fed. Proc.*, **43**, 1633.